# Regional Correlates of Psychiatric Inpatient Treatment

**DOI:** 10.3390/ijerph13121204

**Published:** 2016-12-05

**Authors:** Taina Ala-Nikkola, Sami Pirkola, Minna Kaila, Samuli I. Saarni, Grigori Joffe, Raija Kontio, Olli Oranta, Minna Sadeniemi, Kristian Wahlbeck

**Affiliations:** 1 University of Helsinki and Helsinki University Hospital, Välskärinkatu 12, Helsinki FI-00029, Finland; minna.kaila@helsinki.fi (M.K.); grigori.joffe@hus.fi (G.J.); raija.kontio@hus.fi (R.K.); 2 Unit for Mental Health, National Institute for Health and Welfare (T.H.L.); Mannerheimintie 168, Helsinki FI-00270, Finland; minna.sadeniemi@thl.fi (M.S.); kristian.wahlbeck@thl.fi (K.W.); 3 Public Health Medicine, University of Helsinki and Helsinki University Hospital, Helsinki FI-000014, Finland; 4 University of Tampere School of Health Sciences, and Tampere University Hospital, Lääkärinkatu 1, Tampere FI-33014, Finland; sami.pirkola@staff.uta.fi; 5 Turku University Hospital and University of Turku, Kiinanmyllynkatu 4-8, Turku FI-20520, Finland; samuli.saarni@tyks.fi (S.I.S.); olli.oranta@tyks.fi (O.O.); 6 University of Turku, Turku FI-20014, Finland; 7 Department of Social Services and Health Care, City of Helsinki, Helsinki FI-00099, Finland; minna.sadeniemi@hel.fi

**Keywords:** psychiatry, mental health services, hospitalization, integrative medicine

## Abstract

Current reforms of mental health and substance abuse services (MHS) emphasize community-based care and the downsizing of psychiatric hospitals. Reductions in acute and semi-acute hospital beds are achieved through shortened stays or by avoiding hospitalization. Understanding the factors that drive the current inpatient treatment provision is essential. We investigated how the MHS service structure (diversity of services and balance of personnel resources) and indicators of service need (mental health index, education, single household, and alcohol sales) correlated with acute and semi-acute inpatient treatment provision. The European Service Mapping Schedule-Revised (ESMS-R) tool was used to classify the adult MHS structure in southern Finland (population 1.8 million, 18+ years). The diversity of MHS in terms of range of outpatient and day care services or the overall personnel resourcing in inpatient or outpatient services was not associated with the inpatient treatment provision. In the univariate analyses, sold alcohol was associated with the inpatient treatment provision, while in the multivariate modeling, only a general index for mental health needs was associated with greater hospitalization. In the dehospitalization process, direct resource re-allocation and substituting of inpatient treatment with outpatient care per se is likely insufficient, since inpatient treatment is linked to contextual factors in the population and the health care system. Mental health services reforms require both strategic planning of service system as a whole and detailed understanding of effects of societal components.

## 1. Introduction

Mental health and substance abuse services (MHS) across the globe are undergoing changes in governance, structure, and content [1,2,3]. Since the 1990s, there has been a major shift from institutional care towards outpatient community care, with the aim of creating balanced care models [4,5,6] and alternatives for acute inpatient care [7,8,9]. The exclusive use of specialized health care has been associated with lower quality of life and fewer social contacts and networks [10]. The reasons for hospitalization are linked with affective symptoms, suicidal intoxication, and poor medication compliance in psychosis patients [11]; social withdrawal and conflicts with family members are also often reasons for hospitalization [12]. Severe mental health illnesses like schizophrenia are associated with longer hospitalizations in general [9,13,14]. In primary care, social networking can be supported differently, so joint use of specialized and primary care has been recommended [10].

How community and hospital care are best combined depends on specific local circumstances related to mental health policy and existing service structures [10,15,16]. According to the World Health Organization’s (WHO) Mental Health Action Plan 2013–2020, policy level decision-making and strong leadership are needed to accomplish a transformation [1].

Finland is a sparsely populated country of 5.5 million inhabitants, where municipalities are responsible for social and health care services. Each municipality is free to provide the public services as a municipal activity, or to purchase the services from an external provider, e.g., another municipality, a joint municipal authority or even a private provider. For specialized public health services, including specialized mental health care, the municipalities join to form hospital districts. Finland is divided into 21 hospital districts. The municipal health centres are the main providers of primary care services, and the hospital districts, which are owned and governed by the municipalities, are the main providers of specialist services. Current reforms in Finland are aimed at a profound integration of the primary and specialized mental health as well as social services (altogether further referred to as MHS) [17].

Likewise, in Finland, the MHS strategy 2010–2015 is aimed at developing more patient-centered, community-based and integrated psychiatric and somatic services, while further limiting the use of hospital-based services [18,19,20,21]. Despite the policy of investing in community care and the deinstitutionalization process, most MHS resources in Southern Finland are still allocated to hospital and non-hospital residential services, while low-threshold outpatient services are scarce [21,22,23].

Previously we reported that MHS diversity positively correlates with catchment area population size. We also noted that catchment areas with an outpatient-based MHS structure have fewer personnel (in full-time equivalents) than areas with hospital-centered services [20]. These findings have raised interest in the interrelationships between community-oriented care and inpatient care. In essence, it is important to understand which factors in the MHS structure and its client pool explain the currently large variation in psychiatric hospital use. For the purposes of the current reforms, including an ongoing dehospitalization process with increasing outpatient care and the integration of psychiatry with general hospitals, more knowledge on the preconditions for reducing the relatively high level of psychiatric hospitalization is needed [19].

We hypothesized that more diverse structure in mental health services with a relative emphasis on outpatient and day care services would be associated with a reduced number of inpatient treatments in acute and non-acute psychiatric facilities [4].

The aim of the present study is to explore whether the MHS structure (diversity of services and balance of personnel between community and hospital care) and indicators of mental health needs are associated with the provision of acute and time-limited non-acute psychiatric hospital treatment in a regional comparison. 

## 2. Methods

### 2.1. The Study Area

REFINEMENT (REsearch on FINancing systems’ Effect on the quality of MENTal health care) is a collaborative project of nine EU countries (for the second phase, internationally referred to as CEPHOS-link, six EU countries: Italy, Norway, Romania, Slovenia, Austria, and Finland), currently led by the Finnish National Institute for Health and Welfare (THL) and partly funded by the EC Seventh Research Framework. The study is contributing to the evidence base needed to plan reforms in financing and the integration of the MHS and overall health care system. Details of the FIN-REFINEMENT, the Finnish part of project, have been described previously [20,24,25]. Briefly, in this study, the study area included four hospital districts (regions) in the southernmost part of Finland: the Hospital District of Helsinki and Uusimaa, Kymenlaakso (Carea), Etelä-Karjala (Eksote), and the Hospital District of South-Western Finland [20]. These hospital districts consist of municipalities (*n* = 67) that form 13 non-overlapping catchment areas, each equipped with psychiatric inpatient services. The total population in the study area is 2.3 million people, with 1.8 million adults, which is approximately 43% of the Finnish adult population (aged 18+ years). The adult population varied considerably by catchment area, from approximately 18,200 (Turunmaa) to 500,000 inhabitants (Helsinki).

### 2.2. Data Collection

#### 2.2.1. Structure

We classified MHS by means of the European Service Mapping Schedule (ESMS-R). The ESMS-R allows for a standardized description of the key features of mental health service structures and provision, including those services provided by primary care and social services [20,24,25,26,27,28]. The data collection and instrument has been described previously [20,23,24]. Briefly, mental health services are classified into 89 different “Main Types of Care” (MTC) in the ESMS-R classification. The MTC is the main descriptor of the care function (e.g., mobile acute team or acute hospital care). The MTC are organized by the “Basic Stable Input of Care” (BSIC); i.e., the organizational units that provide the services (e.g., acute ward or day care center). MTC are allocated to six main branches of the ESMS-R: (1) information for care; (2) accessibility to care; (3) self-help and voluntary help; (4) outpatient care, (5) day care; and (6) residential care [28,29].

In addition, we collected the numbers of admission rates and of days spent in a hospital or residential services in one year as a means to map the inpatient treatment provision in the areas. The data were collected between 2012 and 2014 by trained researchers from public corporation data sources for the years 2012 and 2013, with co-operation from local stakeholders. Data on MHS provided by third sector and private providers were collected by structured questionnaires and followed up where necessary by e-mail and telephone contact.

#### 2.2.2. Service Structure Variables

The diversity of the MHS structure was measured by counting the main types of care (MTC) in outpatient and day care branches, the hypothesis being that diversified outpatient and day services diminish the need for hospital beds. The used terms are definite on Refinement Glossary [30,31]. “Outpatient services: Setting in which mental health services are provided on an outpatient basis, without overnight stay, either mobile (when the facility is capable of being moved to different locations) or fixed (when the person seeking care must travel to a fixed service site). There is contact between staff and service users for some purpose related to management of their condition and its associated clinical and social difficulties. These services are not provided as a part of the delivery of day care services, and they have at least some qualified health care professionals as staff members. Day care: Care provision (i) is normally available to several consumers at a time (rather than delivering services to individuals one at a time); (ii) provide some combination of treatment for problems related to long-term care needs: e.g., providing a structured activity, or social contact and/or support; (iii) have regular opening hours during which they are normally available: and (iv) expect consumers to stay at the facilities beyond the periods during which they have face-to-face contact with staff (i.e., the service is not simply based on individuals coming for appointments with staff and then leaving immediately after their appointments). The care delivery is usually planned in advance”.

**Figure 1:**
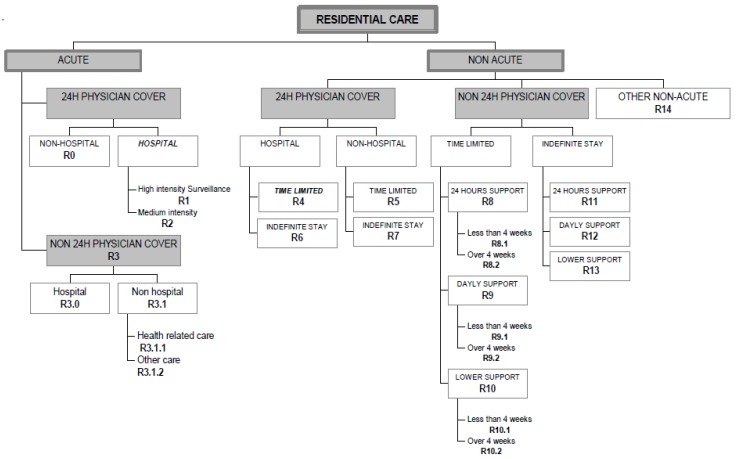
The residential care classification of the ESMS-R tool [28]. Wards included in the study are given in bold.

The ESMS-R tool’s residential service branch includes a total of 21 different MTC (Figure 1). Acute wards are defined by the ESMS-R as high and medium intensity acute care facilities with 24-h physician cover in a registered hospital. Non-acute wards are defined as time-limited facilities where a fixed maximum period of residence is routinely specified (temporary stay). A facility should be classified as time-limited if a maximum length of stay is fixed for at least 80% of those entering the facility [28,30].

As an indicator of acute and semi-acute psychiatric inpatient treatment, we used the total number of used beds on acute psychiatric hospital wards (ESMS-R categories R1 and R2) and non-acute, time-limited psychiatric hospital wards (R4). The calculation of the per capita number of inpatient treatment beds by each catchment area population including the inpatient treatment beds physically located both inside and outside of the catchment areas. We also explored the proportion of beds used at other types of hospital wards (e.g., acute non-physician cover) and non-hospital residential service units (e.g., supported housing).

#### 2.2.3. Socioeconomic and Health Factors Related to Mental Health Needs

The socioeconomic factors included are those commonly related to mental health needs: education index (education years after primary school), alcohol sales (liters of 100% alcohol per person), unemployment rate in the working age population, and the proportion of single-person households (%).

The mental health index (MHI) is an indicator of population mental health status calculated for each catchment area using three years of data on (1) number of suicides and suicide attempts; (2) persons eligible for special reimbursement for antipsychotic medication; and (3) persons on disability pension (18–64 years old) due to mental disorders. The MHI for the whole of Finland is set to 100. An MHI smaller than 100 indicates a better than average state of mental health. The socioeconomic data from 2011–2012 were collected for the period 2012–2013 from Statistics Finland and from the Indicator Bank Sotkanet (www.sotkanet.fi).

#### 2.2.4. Personnel Resource Factors

The allocated personnel full-time equivalents (FTE) per used acute and semi-acute bed indicate how well the wards were resourced, the hypothesis being that better resourcing would lead to shorter hospitalizations. Community orientation was operationalized as the proportion of all personnel allocated to outpatient and day care services, the hypothesis being that better community resourcing would lead to lower hospital use. The personnel allocation for community services is counted as two different variables: (1) the sum of total FTE allocated to outpatient and day care; and (2) the community-based service ratio (outpatient and day care FTEs/divided residential FTEs), where 100% is an equal allocation.

### 2.3. Data Analysis

The SPSS statistics program version 22 was used for the analyses. Scatterplots were used to explore and illustrate the associations between indicators. Spearman correlation analysis was used to investigate the association between acute and semi-acute inpatient treatment provision (independent variable) and the explanatory MHS service structure variables: diversity of outpatient and day care services, personnel FTE allocation per bed, and community orientation. The correlations between acute and semi-acute beds and other hospital beds and non-hospital beds were also analyzed.

The main outcome in the analyses was the provision of acute and semi-acute inpatient treatment, counted by used beds per 1000 (18+) on acute and semi-acute wards. The dependences between the explanatory variables and the provision of inpatient treatment were analyzed using linear regression analysis. First, univariate analyses were performed for each explanatory factor separately. Next, analyses were controlled for MHI. The significance level was set to *p* < 0.05. The analyses were carried out with Statistical Package for Social Sciences (SPSS) version 22 (IBM, Armonk, NY, USA).

## 3. Results

### 3.1. The Inpatient Treatment Provision and the Mental Health Service Structure

#### 3.1.1. Inpatient Treatment Provision and Community (Outpatient and Day Care) Service Diversity

The provision of treatment in different ward levels is presented in Table 1. The provision of acute and semi-acute hospital beds varied four-fold, between 0.27 and 1.00 beds per 1000 adults. The provision of other hospital beds varied from 0.0 to 0.08. The median length of stay was 18.7 days (mean 22.7, SD 7.7, range 20.02).

The highest provision of acute and semi-acute beds was found in the small Turunmaa area (1.00), but notably this is based on only one ward situated in the area, and the per capita total of hospital provision by bed (1.06) is close to the average level per 1000 adults (mean 0.78).

**Figure 2:**
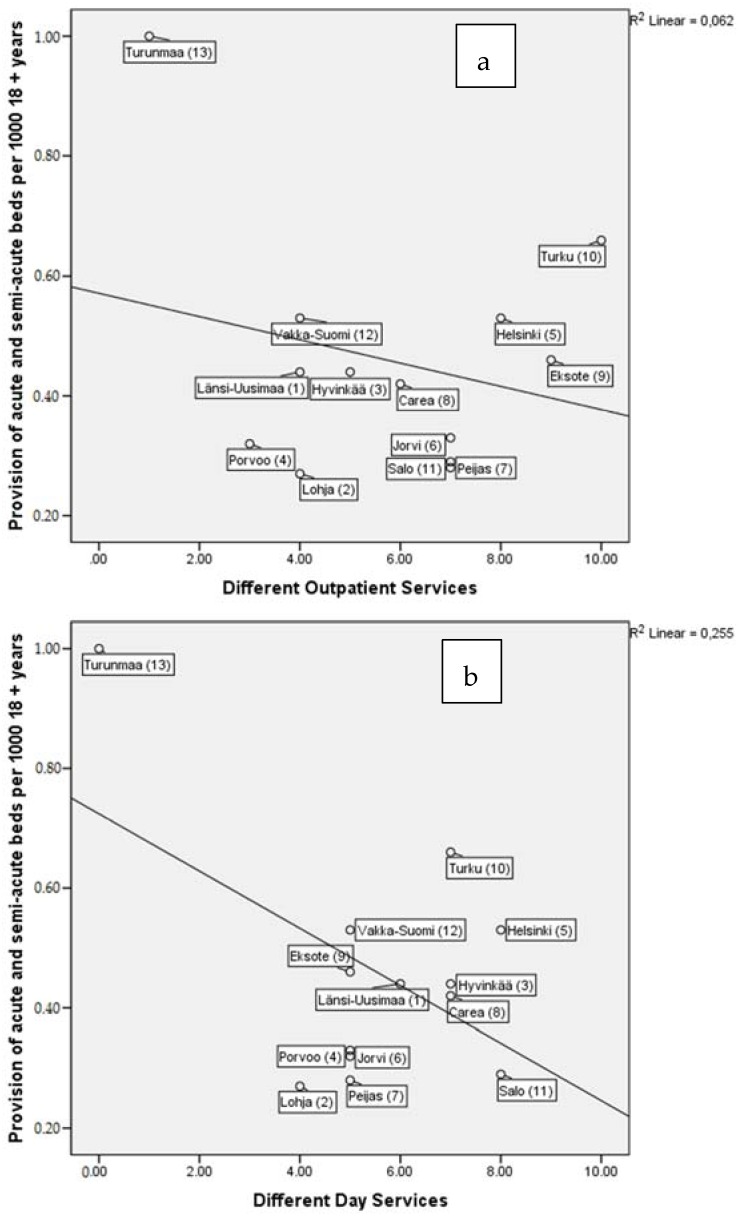

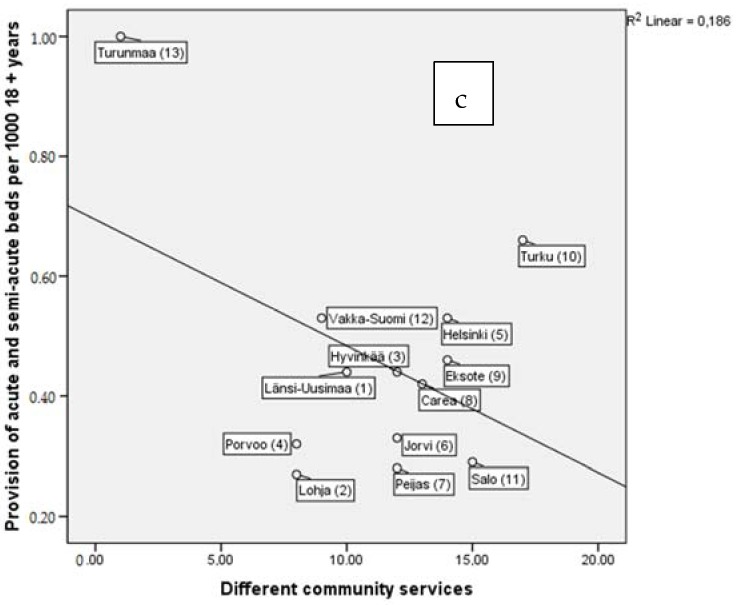
Association between acute- and semi-acute (time-limited) inpatient treatment provision and different; (**a**) outpatient-, (**b**) day care-, and (**c**) total community services- available. The numbers in brackets after catchment areas indicate the data collection order and were used in a previous article [20].

In scatterplots, the smallest district of Turunmaa, which also used the acute ward for long-term care, appeared to be more or less an outlier. Therefore, we also analyze and discuss its role separately where appropriate, while excluding it from all linear regression analyses. Figure 2a–c present the scatterplot and regression line between per capita acute or semi-acute bed provision and the availability of different outpatient and day care and total community (outpatient and day care) services. There was a weak direct relationship between the diversity of day care services and the provision of hospital beds (Figure 2b). The same effect is shown for total community services and the provision of hospital beds (Figure 2c). The service structure of the smallest Turunmaa area, with only one ward for all residential care, affected the model, intensifying the significance (visible in Figure 2a,c). A sensitivity analysis was performed, excluding the Turunmaa area, in which no significant relations were found.

**Figure 3:**
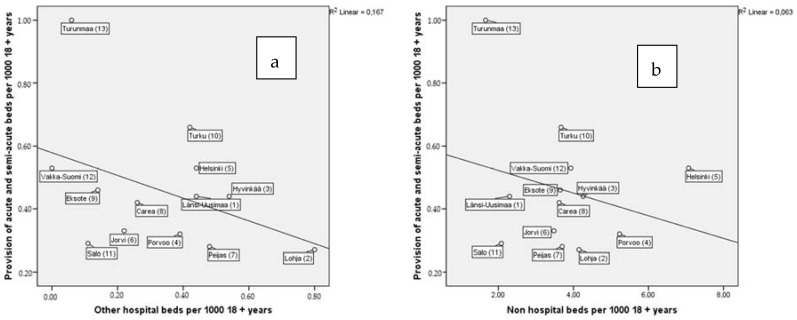
Associations between acute- and semi-acute (time-limited) inpatient treatment provision and; (**a**) other hospital bed- and (**b**) non-hospital bed- provision per 1000 adults (18+). The numbers in brackets after catchment areas indicate the data collection order and were used in a previous article [20].

The provision of other residential services and other hospital beds and non-hospital beds were not significantly associated with acute and semi-acute inpatient treatment provision (Figure 3a,b).

#### 3.1.2. The Inpatient Treatment Provision and Personnel Resource Factors

The highest per capita use of total personnel resources was found in Länsi-Uusimaa (5.1) and the lowest in Jorvi (2.1) (range 2.1–5.1, mean 3.3). The highest per capita use of community-based personnel resources, counted as the sum of outpatient and day care resources, was found in Länsi-Uusimaa (1.9) and the lowest in Turunmaa (0.8) and Lohja (0.9) (range 0.8–1.9, mean 1.3). The grade of community orientation varied from 0.9 to 0.3 (Table 2).

### 3.2. The Inpatient Treatment Provision and Mental Health Need Indicators

There were some differences between catchment areas regarding socioeconomic factors (Table 3). The average MHI was 90.3 (SD 21.9), indicating that in the study area as a whole, the need for mental health services may be lower than in Finland overall. The areas located in the Helsinki and Uusimaa Hospital District, near Finland’s capital, had a lower MHI than the national average. Further away from the capital, i.e., in Kymenlaakso, Etelä-Karjala, and South-Western Finland (Areas 10–13), the MHI was higher than the national average.

In Spearman’s correlation analysis, the sold alcohol in liters per adult was significantly associated with the provision of acute and semi-acute hospital beds (ρ = 0.606, *p* = 0.028). The education indices or number of single person households were not significantly associated with the per capita provision of acute and semi-acute beds (Table 4).

Various service structures and socioeconomic factors were investigated for their association with the inpatient beds provision through linear regression modeling (Table 5). The level of mental health problems in the population was taken into account by adjusting the analysis for MHI. All linear regression modeling was done while excluding the catchment area considered to be an outlier (Turunmaa).

In the linear regression modelling, none of the indicators except MHI were associated with the provision of beds.

## 4. Discussion

We examined a spectrum of factors possibly linked to the per capita level of acute and semi-acute psychiatric inpatient treatment provision by means of a regional comparative analysis. The provision of acute and semi-acute hospital beds was hypothesized to react to changes in acute outpatient service activity. Contrary to our expectations, we found only statistically non-significant signals, indicating that a higher diversity in outpatient and day services might be associated with lower hospital provision. The significance of this finding seemed to be dependent on one distinguishable catchment area, and disappeared when the area was excluded from the linear regression modelling. Regarding socioeconomic characteristics, we found that higher alcohol consumption (measured as sales) was associated with higher acute and semi-acute inpatient treatment provision. Somewhat surprisingly, neither inpatient nor outpatient personnel resources seemed to play a major role in the variation in psychiatric inpatient treatment provision. Still, our study did not find a correlation between the provision of acute of semi-acute beds and any variable describing quality of outpatient care. This suggests that the relationship between improving the quality of outpatient care and reducing hospital care is not straightforward or automatic.

According to the linear regression modeling, mental health needs as indicated by the MHI were associated with the per capita provision of psychiatric acute and semi-acute hospital beds. Altogether, the provision of acute and semi-acute beds is linked to catchment areas’ socioeconomic factors and population mental health need indicators. The association of alcohol sales with inpatient treatment may reflect the effect of socioeconomic correlates, but also the effect of a higher incidence of alcohol-related psychoses [32]. It is possible that the diversity of community care services in the catchment areas studied did not reach the critical level needed to have an impact on the provision of inpatient treatment. This may point to a need for a more diverse setting for acute and mobile services provided at home or in other living circumstances, e.g., supported housing localities. The alternatives for hospitalization in community-based structures are important. Mental health service reform has to be based on research and data, while also taking into account the quality of services in terms of diversity, societal components like socioeconomics, and the estimated needs of the population.

### 4.1. Comparison to Previous Studies

Some previous studies have used ESMS to investigate MHS internationally or nationally. Salvador-Carulla et al. [33] compared mental health systems in Italy and Spain and found great differences related to the pattern of service provision and service use between countries. They suggested developing innovative community services with low hospital bed use, high rates of day services, and contacts with the community. Tibaldi et al. [34] recognized that areas with more intensive use of community services, and fewer people living alone, had lower acute hospital bed occupancy rates, congruent with what we found in our setting. Rezvyy et al. [35], using the ESMS instrument, reported that in rural areas with inadequate community-based services, patients are more often admitted to hospital care. In our study, we did not find any association between the community-based services, counted as FTE allocation or diversity of services, and the provision of acute and semi-acute hospital beds. The difference between the results might be explained by the level of available community-based services, since in our study no catchment area was found to lack community services, contrary to the situation in the study of Rezvyy et al. [35], where some patients could not be discharged due to a lack of local services.

In the present study, the MHI was correlated with the provision of acute and semi-acute beds at a catchment area level. This is in line with our previous study, where the psychiatric hospital admission rate was correlated with the MHI and socioeconomic indicators in municipal-level comparisons [25,36]. This suggests that the MHI is a robust indicator of mental health care needs, and we support its use as a controlling variable when searching for differences in health system performance. 

Our findings on alcohol sales and inpatient treatment are supported by previous national and international findings [37,38]. Previously, in a large Finnish register (TERVEYS 2000) study, the lifetime prevalence of AIPS (alcohol-induced psychotic syndrome) was 0.5% of the survey participants. In their lifetime, all participants with AIPS had had some mental health or alcohol treatment contact and 82.1% had psychiatric hospital treatment [32,39]. Instead, the lifetime prevalence of other-than-alcohol substance abuse psychoses in Finland is relatively low in the population aged over 30 years [39]. This is line with our previous findings with the same dataset, where many explanatory socioeconomic indicators, e.g., unemployment, alcohol consumption, and single households, intercorrelate significantly [20].

Previously Myklebust et al. [40] reported that in Norway outpatient and day-hospital services may be filters in the pathway to inpatient care. They underlined that this depends on the structure of the whole service system, since decentralized psychiatric beds may hinder the development of various local psychiatric services.

Community-based alternatives to inpatient treatment, such as day hospitals, have been found to be effective [41], but there are patient groups that need more intensive care. Especially in cases of schizophrenia or other severe mental disorders, there may be a lack of effective, active, and assertive treatment in the community [42]. There are some previous studies that suggest that residential alternatives, such as community-based crisis services, provide an alternative to hospital treatment [43,44]. There are also substance abuse (including all kind of drugs and alcohol) users who need a high number of both inpatient and outpatient services and therefore integrative care [45,46]. The severe symptoms as well as the lack of social support and community services in addition to accommodation problems might prolong the length of the hospital stay when a more active and assertive community treatment would likely reduce readmission [46]. The community-based structure also seems to be better than hospital-based treatment in terms of the outcome and quality of life for individuals with chronic mental disorders [16].

Harris et al. [47] quantified the need for hospital services in Australia based on the prevalence of severe mental disorders, national and international benchmarking data, and trend data from service utilization; based on this analysis, they recommended 20 acute and 10 non-acute beds per 100,000 inhabitants. Comparable figures for the inpatient treatment provision in our study area would be 30 acute and 18 non-acute (semi-acute) beds (48 beds per 100,000), which is 62% more beds than recommended in the Australian context.

In addition to the structural deinstitutionalization of mental health care, there has been a call for greater service coordination between inpatient and outpatient services [40]. Intensive case management can reduce the provision of hospitalization if the hospitalization level is high, but the effect is limited if inpatient treatment is already at a low level [7]. Intensive case management methods like assertive community treatment are useful, especially regarding severe mental health illnesses [48]. In general, modern mental health strategies promote the role of primary care and public mental health [49]. These elements are recommended when seeking to encourage continued efforts to develop modern, flexible, and versatile community services while limiting hospitalization.

### 4.2. Strengths and Limitations

The strengths of this study include the use of an internationally validated instrument, the ESMS-R, in the classification and comparison of services. We used ESMS-R for mapping [28,33] and national register-based sociodemographic data to indicate the mental health service needs of the population. The ESMS-R instrument is a valid instrument for MHS classification and comparison.

The strength of the ESMS-R is that the comparison includes the full coverage of MHS in the study area, including primary, secondary, and tertiary care, as well as social services and voluntary services. Data were collected by trained persons in strong cooperation with local stakeholders.

There are some limitations to the study. The relatively small number of catchment areas (*n* = 13) offers limited possibilities for statistical analysis of the differences between the areas. Due to the limited sample size and consequential lack of statistical power, there may be undetected dependencies between inpatient treatment and explanatory variables. Furthermore, one area appeared to have very specific service structure characteristics, raising concerns about its comparability. For that reason, explorative analyses were used. The data were collected from consecutive years (2012–2013), so some rapid reductions in inpatient treatment and restructuring developments during this period will not be seen in the catchment areas (1–9) where the first data collection was done. Due to missing patient episodic data, the average length of stay is not evaluated further. For the same reason, the provision of inpatient treatment is compressed at the median level, counted by dividing the total hospital days by the number of admissions. There would be differences between diagnosis groups in terms of how inpatient treatment provision was hindered, based on data limitations. In our study areas, there is to a large extent homogeneity and consistency in the legal and steering structures. However, there were some differences between the areas regarding the MHS provided by the municipalities or hospital district or in combinations of those two.

## 5. Conclusions

The relationship between population mental health needs, as indicated by the MHI, and inpatient treatment provision was confirmed by our study. Other indicators of need were not significant after controlling for the MHI, supporting its value in predicting need. This could be interpreted positively, meaning that objective needs guide the system to some extent. Based on our study, THL-MHI is a relevant indicator for planning and evaluating the MHS structure and especially for evaluating the inpatient treatment provision, at least for severe mental health illnesses.

Direct re-allocation of resources from inpatient treatment to outpatient care is unlikely to be an adequate mental health service reform on its own; the reform should rather be based on research and data, while taking into account the quality of services in terms of diversity, societal components like socioeconomics, as well as the estimated needs of the population. Improvement efforts for outpatient care need to be accompanied by specific, locally tailored, and context-sensitive efforts to reduce hospitalized care.

## Figures and Tables

**Table 1:** Provised beds on different types of psychiatric wards and non-hospital services per 1000 (18+) and community service diversity.

**Catchment Area**	**Länsi-Uusimaa (1)**	**Lohja (2)**	**Hyvinkää (3)**	**Porvoo (4)**	**Helsinki (5)**	**Jorvi (6)**	**Peijas (7)**	**Carea (8)**	**Eksote (9)**	**Turku (10)**	**Salo (11)**	**Vakka-Suomi (12)**	**Turunmaa (13)**	**SD**	**Weighted Mean**
Size of catchment area 18+ population	35,296	70,379	139,734	74,611	501,929	230,005	187,332	143,265	109,379	151,616	128,039	81,392	18,200		
Beds on acute and semi-acute wards (R2, R4) *	0.44	0.27	0.44	0.32	0.53	0.33	0.28	0.42	0.46	0.66	0.29	0.53	1.00	0.20	0.45
Beds on other hospital wards (R3, R6)	0.44	0.80	0.54	0.39	0.44	0.22	0.48	0.26	0.14	0.42	0.11	0.00	0.06	0.22	0.33
Beds on non-hospital services **	2.30	4.15	4.26	5.23	7.07	3.48	3.70	3.62	3.65	3.67	2.08	3.93	1.65	1.38	3.75
Total beds in all categories	3.18	5.22	5.24	5.94	8.04	4.03	4.46	4.30	4.25	4.75	2.48	4.46	2.71	1.44	4.54
Community service diversity ***	10	8	12	8	14	12	12	13	14	17	15	9	1		

* R1 Wards were not found; ** ESMS-R codes: R0, R3.1, R3.1.1, R3.1.2, R5, R7, R8, R8.1, R8.2, R9.1, R9.2, R10.1, R10.2, R11, R12, R13, R14; *** Different services (main types of care) on community services (outpatent and day care services).

**Table 2:** Personnel full time equivalents (FTE) resources on mental health services per 1000 (18+).

Catchment Area	Länsi-Uusimaa (1)	Lohja (2)	Hyvinkää (3)	Porvoo (4)	Helsinki (5)	Jorvi (6)	Peijas (7)	Carea (8)	Eksote (9)	Turku (10)	Salo (11)	Vakka-Suomi (12)	Turunmaa (13)	Study Area
Size of catchment area 18+ population	35,316	70,192	138,973	74,079	497,814	227,605	185,984	141,085	107,612	151,616	128,039.2815	81,392	18,200	1,857,907
Personnel FTE resources per 1000 (18+)														
Day care (D) FTE per 1000	0.6	0.2	0.4	0.3	0.3	0.1	0.1	0.2	0.6	0.4	0.4	0.3	0.0	0.3
Outpatient care (O) FTE per 1000	1.3	0.7	0.9	0.7	1.1	0.9	0.9	0.9	0.8	1.4	1.2	1.0	0.8	1.0
Residential care (R) FTE per 1000	3.2	2.6	2.9	1.8	2.0	1.1	1.5	2.8	1.6	2.9	1.7	2.4	2.2	2.0
Community-based services = FTE D + O per 1000	1.9	0.9	1.3	0.9	1.4	1.0	1.0	1.1	1.4	1.8	1.6	1.2	0.8	1.3
Community orientation (FTE ratio outpatient/residential)	0.6	0.3	0.5	0.5	0.4	0.9	0.7	0.4	0.9	0.4	0.5	0.3	0.3	0.5
Total FTE per 1000	5.1	3.5	4.2	2.7	3.4	2.1	2.6	4.0	3.1	4.7	3.3	3.6	3.0	3.3

FTE: full-time equivalent. The numbers in brackets after catchment areas indicate the data collection order and were used in a previous article [20].

**Table 3:** Socioeconomic factors and population mental health status.

Catchment Area	Population (18+)	Mental Health Index (Not Age Adjusted)	Education Index	Unemployment %	Sold Alcohol (100%) ltr per Person	Single Households (%)
Länsi-Uusimaa (1)	35,296	82.0	3.0	7.2	8.8	40.1
Lohja (2)	70,379	84.5	3.2	7.1	8.0	34.8
Hyvinkää (3)	139,734	72.9	3.5	6.0	7.3	34.2
Porvoo (4)	74,611	73.5	3.3	7.1	7.2	35.2
Helsinki (5)	501,928	83.9	4.1	7.5	9.4	49.0
Jorvi (6)	230,005	65.9	4.6	5.5	6.3	34.4
Peijas (7)	187,332	78.0	3.4	8.0	8.1	38.1
Carea (8)	143,210	110.8	3.0	12.2	8.6	43.9
Eksote (9)	107,612	104.7	3.0	11.8	9.4	43.6
Turku (10)	151,616	144.9	3.7	12.9	8.6	51.4
Salo (11)	128,039	103.8	3.2	8.8	6.5	37.2
Vakka-Suomi (12)	81,391	100.7	3.2	7.1	7.8	35.7
Turunmaa (13)	18,199	101.6	3.2	6.0	9.5	37.5
SD	122,760	19.9	0.5	2.5	1.1	5.7
Mean	143,796	92.2	3.4	8.2	8.1	39.6

Data Statistics Finland ^R^THL, SOTKAnet Statistics and Indicator Bank. Areas 1–9 data from 2011 and Areas 10–13 from 2012; Mental health index from 2012 (including data from 2010–2012); Education years after primary school, e.g., high school, vocational school and university. The numbers in brackets after the catchment areas indicate the data collection order and were used in a previous article [20].

**Table 4:** Correlations between provision of acute and semi-acute beds (per 1000 18+), socioeconomic factors, need indicators and service structure.

Spearman’s Rho (*n* = 13)		Size of Population (18+)	Mental Health Index	Education	Unemployment	Sold 100% Alcohol	Single Households	Different Outpatient Services	Different Day Services	Different Community Services (O + D)	Community Orientation *
Provision of acute and semi-acute beds per 1000 (18+)	Correlation Coefficient	−0.055	0.377	0.163	0.017	0.606 *	0.455	0.092	0.081	0.113	−0.191
	Sig. (2-tailed)	0.858	0.204	0.595	0.957	**0.028**	0.119	0.765	0.792	0.714	0.532

* Community/residential full time equiavalents relation. The numbers in brackets after catchment areas indicate the data collection order and were used in a previous article [20]. The data in bold: significance *p* > 0.05.

**Table 5:** Linear regression models explaining use of acute and semi-acute beds with explanatory indicators. (one by one) standardized by mental health index *.

Explanatory Indicators	Unstandardized Coefficients		*t*	Sig.
	B	Std. Error		
Mental health index (non standardized)	0.004	0.001	2.789	0.021
Size of Population (18+)	3.094 × 10^7^	0.000	1.304	0.224
Education index	0.073	0.061	1.194	0.263
Un-employment	−0.020	0.025	−0.807	0.440
Sold alcohol	0.042	0.030	1.428	0.187
Single households	0.011	0.006	1.774	0.110
Different outpatient services	0.007	0.017	0.390	0.706
Different day services	0.018	0.024	0.736	0.480
Different community services (outpatient and day services)	0.005	0.014	0.353	0.732
Total personnel per acute and non acute time limited bed	−0.094	0.080	−1.169	0.272
Community orientation ratio (community/residential FTE)	−0.026	0.163	−0.159	0.877
Other hospital beds per 1000	−0.024	0.148	−0.159	0.877
Non-hospital beds per 1000	0.037	0.022	1.708	0.122
Total residential beds per 1000	0.035	0.020	1.801	0.105

Dependent Variable: Used acute and non acute time-limited beds per 1000 18+ Catchment area Turunmaan excluded by outlier; * Mental health index (MHI) as only independent variable, other models controlled for MHI.
